# Theoretical study on the insertion reaction of the stannylenoid H_2_SnLiF with X-H bonds (X = N, O, F)

**DOI:** 10.55730/1300-0527.3671

**Published:** 2024-05-02

**Authors:** Shuo WU, Bingfei YAN, Shaoli LIU, Wenzuo LI

**Affiliations:** Department of Applied Chemistry, School of Chemistry and Chemical Engineering, Yantai University, Yantai, P. R. China

**Keywords:** H_2_SnLiF, X-H, M06-2X, QCISD, insertion reaction

## Abstract

The insertion reactions of p-complex (RP) and three-membered ring configuration (RS) of stannylenoid H_2_SnLiF with NH_3_, H_2_O and HF have been studied theoretically by quantum chemical calculation. The structures of reactants, precursors, transition states, intermediates and products have been fully optimized at the M06-2X/def2-TZVP level. The single point energy of all fixed points were calculated using the QCISD method. The calculation results show that the three-membered ring configuration is easier to conduct the insertion reaction. Comparing the reaction energy barriers of RP, RS to NH_3_, H_2_O and HF, we found that the difficulty of the insertion reaction is NH_3_ > H_2_O > HF. The solvent corrected calculation results show that in THF, the reaction energy barrier of RP is lower than that in vacuum, while the reaction energy barrier of RS is higher. This work provides theoretical support for the reaction properties of stannylenoid.

## 1. Introduction

Tetrylenoids R_2_EXM (E = Si, Ge and Sn; X = electronegative group; M = alkali metal) are heavier homologues of carbenoid compounds [1–2], which have electrophilic and nucleophilic properties just like carbenoid. Silylenoids and germylenoids have been successfully synthesized and isolated over decades of exploration, and their structures and reaction properties have been well investigated experimentally and theoretically [3–26]. For example, in 2006, Molev et al. [12] used X-ray crystal diffraction to determine the structure of the first successfully separated fluorolithium silylenoids. From 2010 to 2015, Cho et al. conducted experimental studies on the addition reaction of lithium containing silylenoids with ketones [13], aldehydes [14], and olefins [15]. In terms of theoretical calculations, in 1980, Clark et al. [16] firstly performed theoretical exploration of H_2_SiLiF. In 2014, Qi et al. [17] studied the structural properties of unsaturated silylenoids HP = SiLiF. In 2016, Yildiz et al. [18] made a theoretical study on the synthesis and rearrangement reaction of cyclic silylenoids. For germylenoids, in 2016, Suzuki et al. [19] synthesized and separated stable chlorine containing germylenoids. In 2007, Ma et al. [20] explored the structure and solvation effect of H_2_GeLiF through theoretical research. Then Li et al. explored the structure and properties of silylenoids containing metal Be [21] and Al [22], respectively. These investigations have promoted people’s understanding of the structure and reaction properties of tetralenoids.

Stannylenoid is a class of tetrylenoids with the structural general formula R1R2SnXM, which is an active intermediate. After decades of research, stannylenoid has attracted more and more attention in the field of organotin chemistry, and a large number of experimental studies have also been carried out. Grugel [27] team was the first to predict the existence of stannylenoid compounds in the reaction of aldehyde with some stannylene precursors. Then Arif et al. [28] and Ochiai et al. [29] successfully isolated the stannylenoid compounds and observed their structures by X-ray diffraction. Yan et al. [30] used the corresponding stannylenes to react with cesium fluoride to synthesize stable stannylenoid for the first time. Then Gross et al. [31] found that free stannylene can be obtained through stannylenoid. Subsequently, some experiments also investigated stannylenoid [32,33].

The reactions of silylenoids and germylenoids have been extensively studied such as the insertion reaction [34–50]. However, there are few theoretical research on the structure and reaction of stannylenoids. We think it is urgent to study the structure and reaction of stannylenoids theoretically, which is conducive to clarifying the reaction mechanism. In this work, we carry out systematic theoretical research on the insertion reaction of H_2_SnLiF with NH_3_, H_2_O and HF. We hope this work would supplement the reactivity of stannylenoid and provide a theoretical basis for the reaction of Sn-like compounds.

## 2. Theoretical methods

The M06-2X [51] methods with the def2-TZVP basis set were used to optimize the geometries of fixed points on the potential energy surface, and then the harmonic vibration frequency was calculated at the same level of theory to represent the minimum or first order saddle point of the optimized geometry. In order to improve the processing of electronic correlation, the QCISD [52] method was used to calculate the single point energy of all fixed points at the def2-TZVP theoretical level. The mechanism of the insertion reaction was verified by the analysis of the intrinsic reaction coordinate (IRC) of the possible transition state (TS). In order to consider the solvent effect, the SMD model [53] was used to calculate the geometry and energy of the fixed point in the insertion reaction, and tetrahydrofuran (THF) solvent was selected (dielectric constant ɛ = 7.4257). The Multiwfn and VMD softwares were used to draw frontier molecular orbitals [54–55]. All calculations were performed using the Gaussian 09 [56] series of programs.

## 3. Results and discussion

The previous work [57] has shown that when stannylene H_2_Sn: and alkali halide LiF coexist, they are difficult to separate from each other and will exist in the form of stannylenoid H_2_SnLiF. The most stable configuration of H_2_SnLiF at HF/3-21G level was found to be the p-complex configuration, followed by the three-membered ring configuration, and the energy barrier for the conversion between the two configurations is relatively large and difficult to convert. The M06-2X/def2-TZVP calculations in present work predicated similar results with Ref. [57]. There are three types of structures, namely p-complex configuration (RP), three-membered ring configuration (RS) and “classical” tetrahedral configuration for H_2_SnLiF, which energies at the QCISD/def2-TZVP//M06-2X/def2-TZVP level are −322.1117, −332.0977 and −322.0687 a.u., respectively. Therefore, the p-complex configuration and three-membered ring configuration are two basic structures of H_2_SnLiF. On the other hand, the energy barrier for RP to RS is 111.96 kJ/mol, and the energy barrier for RS to RP is 75.62 kJ/mol, which means it is difficult to convert into each other for RP and RS. Therefore, in this work, we use the p-complex configuration (RP) and three-membered ring configuration (RP) to explore the insertion reaction with NH_3_, H_2_O, and HF.

The insertion reaction of stannylenoid H_2_SnLiF with X-H bonds (X = N, O, F) can be described as the following equation:


$${\text{H}}_{2}\text{SnLiF}+{\text{XH}}_{\text{n}}\to {\text{H}}_{3}{\text{SnXH}}_{\text{n-}1}+\text{LiF}(\text{X}=\text{N},\text{O},\text{F},\text{n}=3,2,1)$$

**Figure 1:**
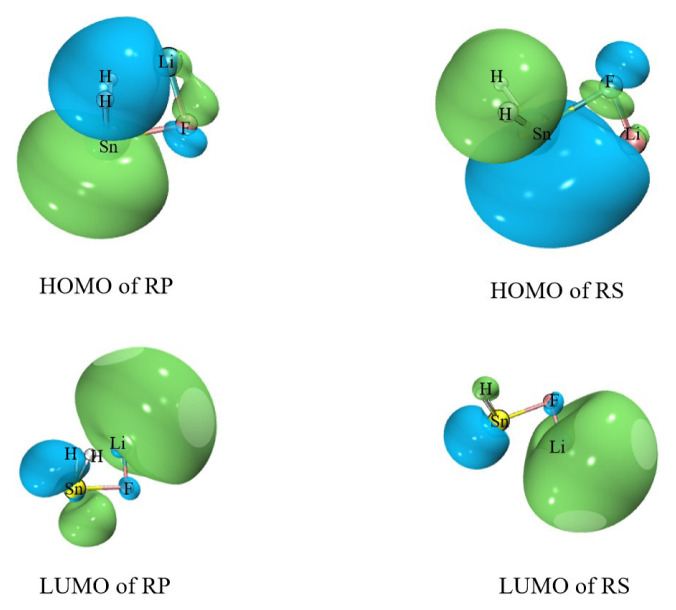
Frontier molecular orbitals of p-complex configuration (RP) and three-membered ring configuration (RS) of H_2_SnLiF calculated at M06-2X/def2-TZVP level.

**Figure 2:**
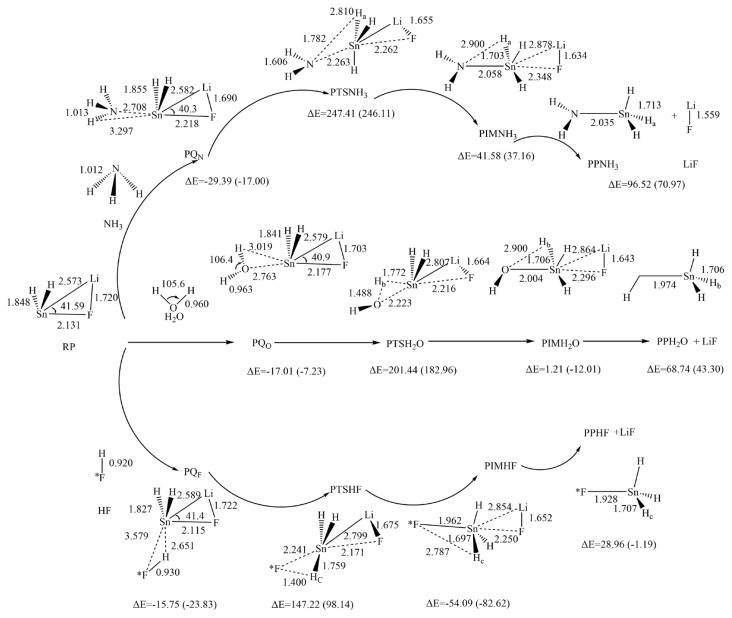
Schematic diagram of relative energy and structural parameters of each stationary point of the insertion reaction channel between RP and NH_3_, H_2_O and HF and the reaction potential energy surface under the M06-2X method (the values in parentheses are the results obtained in THF solvent, bond length is in Å, bond angle is in degree).

**Figure 3:**
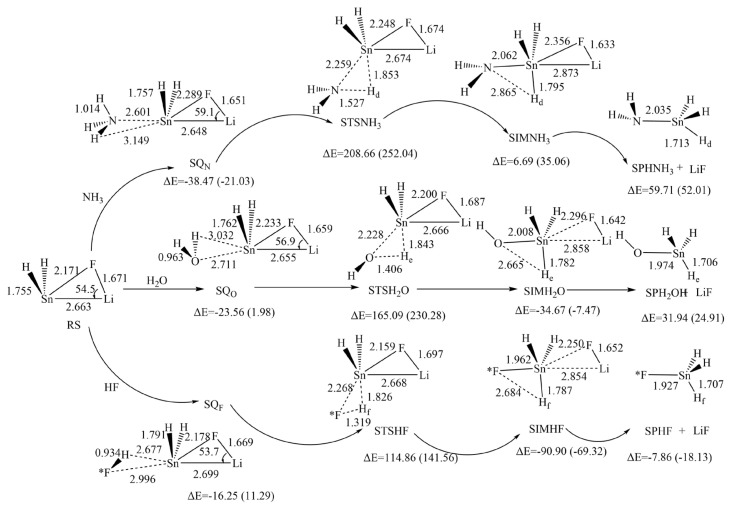
Schematic diagram of relative energy and structural parameters of each stationary point of the insertion reaction channel between RS and NH_3_, H_2_O and HF and the reaction potential energy surface under the M06-2X method (the values in parentheses are the results obtained in THF solvent, bond length is in Å, bond angle is in degree).

**Figure 4:**
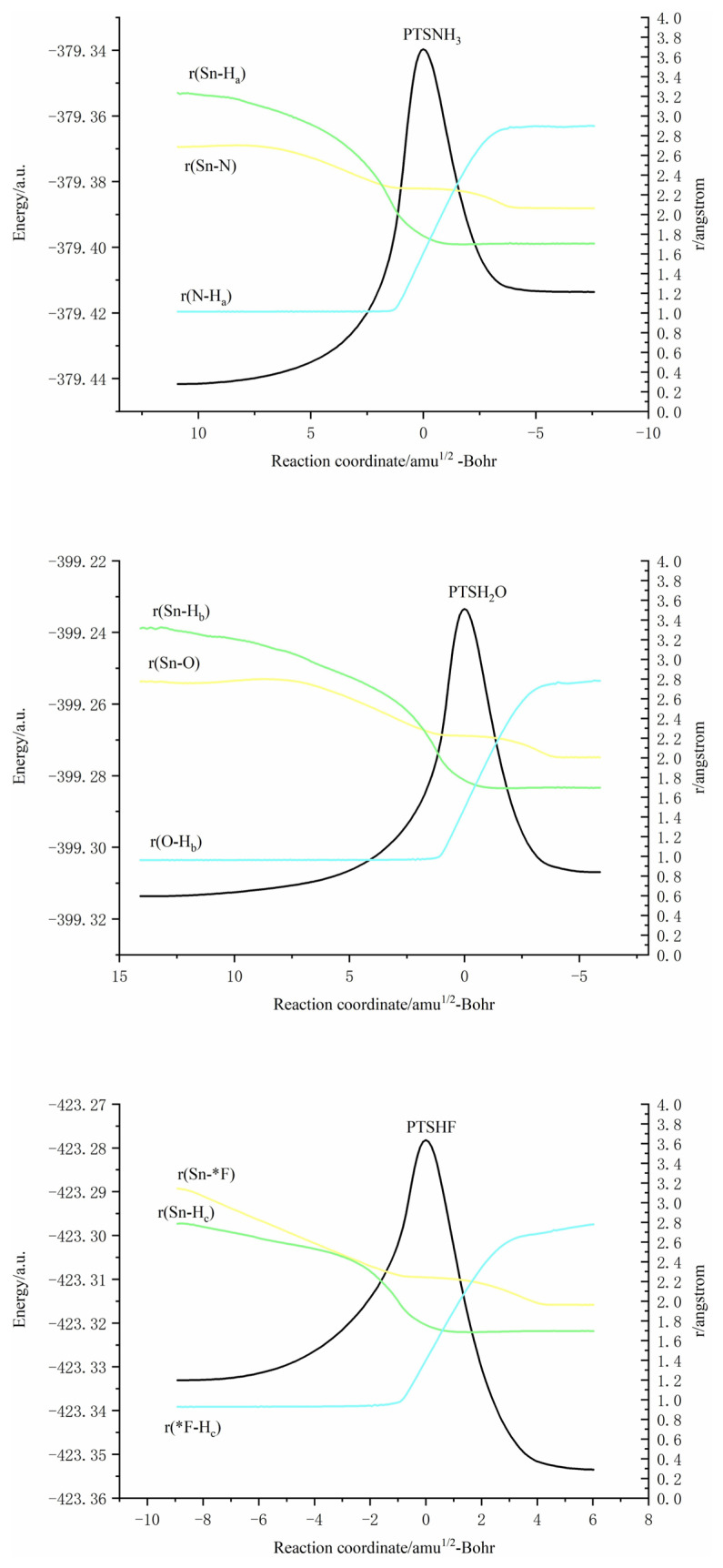
Changes in energy and bond distance of reaction coordinate of RP and XH_n_ (X = N, O, F, n = 3, 2 1) insertion reaction.

**Figure 5:**
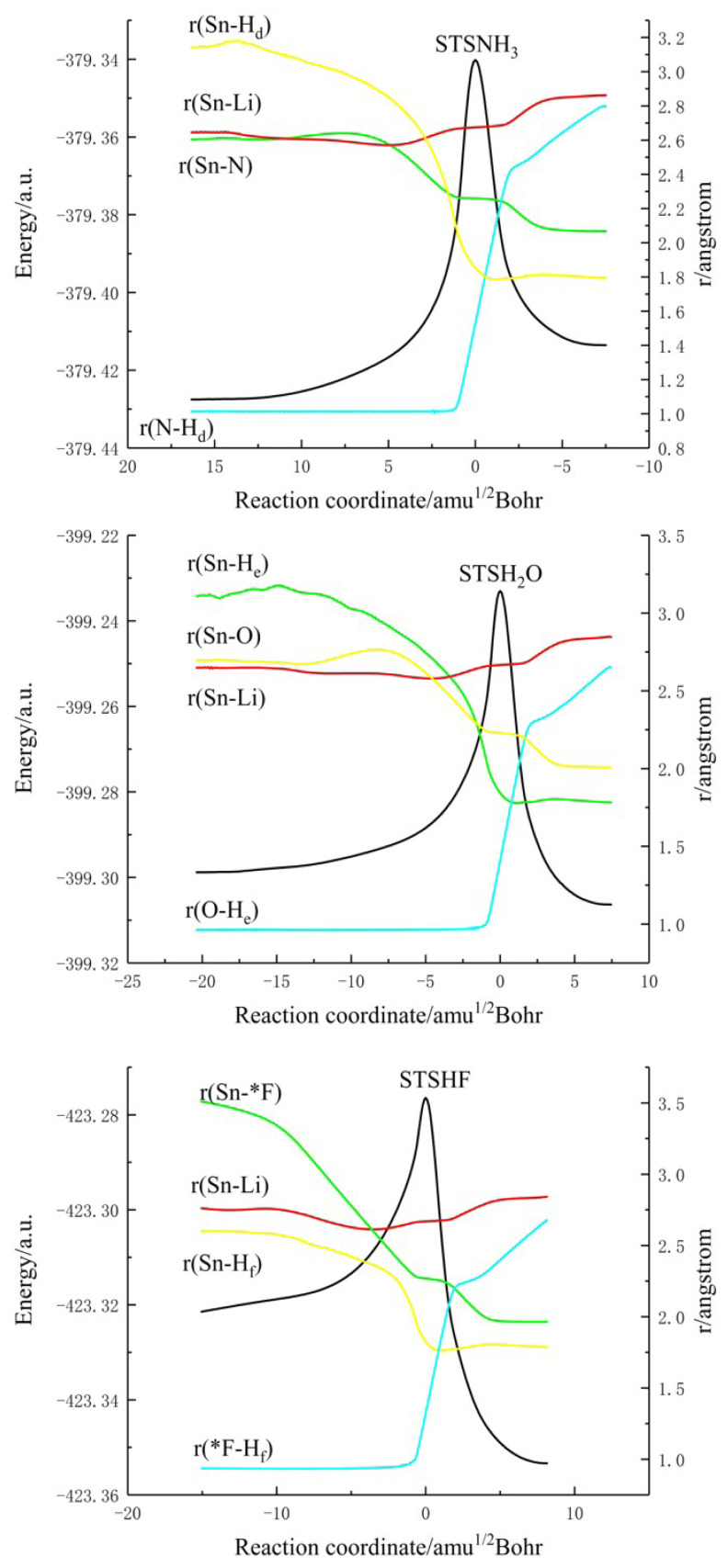
Changes in energy and bond distance of reaction coordinate of RS and XH_n_ (X = N, O, F, n = 3, 2, 1) insertion reaction.

Based on the calculated results, it can be reasonably predicted that the first step of the reaction between H_2_SnLiF and XH_n_ is to form the precursor complex (Q). And there is a transition state (TS) and an intermediate (IM) along the reaction potential energy surface, and these structures connect reactants and products respectively. The Figure 1 shows the frontier molecular orbitals of p-complex configuration (RP) and three-membered ring configuration (RS) of H_2_SnLiF. The Figures 2 and 3 show the reaction process and the relative energy and structural parameters of each stationary point of the potential energy surface (relative to the corresponding reactants). Figures 4 and 5 show changes in energy and bond distance of reaction coordinate of RP and RS with XH_n_ insertion reaction. Tables S1–S6 in Supporting information shows the charge changes of each atom in the insertion reaction of RP, RS with XH_n_, respectively. Table S7 and S8 in Supporting information shows the energy of each stationary point on the potential energy surface of RP and RS insertion reaction.

### 3.1. Insertion reaction of p-complex configuration (RP)

The p-complex structure (RP) of H_2_SnLiF can be regarded as a stannylene complex with ionic compound LiF (Figure 1). RP is formed by contributing some electrons of F atom in LiF to the unoccupied p orbital of Sn atom in singlet H_2_Sn. In fact, there is also weak interaction between Li atom (with positive charge) and two H atoms (with negative charge). The highest occupied molecular oibital (HOMO) of RP is mainly composed of σ orbital on Sn atoms, so the RP shows obvious nucleophilicity in the σ orbital direction. The insertion reaction between RP and X-H bonds is caused by the interaction between the σ orbitals occupied by Sn atom and the s orbitals on H atoms of X-H bonds. When the H atom further approaches the Sn atom through the electrostatic interaction, the X end of the X-H bond also interacts with the Sn atom.

#### 3.1.1. The structures and energies of the precursor complexes

When XH_n_ is close to RP, the H end of X-H bonds and the σ orbital of Sn atom interaction forms precursor PQ_N_, PQ_O,_ and PQ_F_. It can be seen from Figure 2 that the H_2_SnLiF part of precursor PQ_N_, PQ_O,_ and PQ_F_ structure has little change compared with the reactant. The Sn-H distances are 3.297, 3.019, and 2.651 Å, and the distances of Sn-N, Sn-O and Sn-*F are 2.708, 2.763, and 3.579 Å, respectively. The relative energies (shown in Figure 2) of PQ_N_, PQ_O_ and PQ_F_ are −29.39, −17.01, and −15.75 kJ/mol, respectively.

#### 3.1.2. The structures and energies of the transition states

As XH_n_ further approaches RP, the interaction between XH_n-1_ and the p orbital of Sn atom reaches the transition state RTSXH_n_. As shown in Figure 2, the bond lengths of Sn-H_a_, Sn-H_b,_ and Sn-H_c_ are 2.810, 1.772, and 1.759 Å, and the distances of Sn-N, Sn-O and Sn-F are 2.263, 2.223, and 2.241 Å respectively. Compared with PQ_X_, the bond lengths of Sn-H and Sn-X are significantly shorter. It can be seen from Tables S1–S3 in Supporting information that in this process, the natural charge of Sn atom increased from 1.438 to 2.242, 2.288, and 2.288, when X = N, O, F, respectively, and the natural charge of X and H atoms decreased significantly, which indicates that RP showed nucleophilic behavior in insertion reaction. Frequency analysis calculations performed at the M06-2X/def2-TZVP level of theory show that PTSX_n_ has a unique imaginary frequency (1413.7, 1438.8, 1393.9 i cm^−1^). IRC analysis shows that PTSX_n_ is the real transition state in the insertion reaction of H_2_SnLiF with XH_n_, and correctly connects the precursor complex PQ_X_ and the intermediate PIMXH_n_. The relative energies of PTSXH_n_ are 247.41, 201.44, and 147.22 kJ/mol, when X = N, O, F, then the potential energy barrier of the insertion reaction are 276.80, 218.45, and 162.97 kJ/mol, respectively.

#### 3.1.3. The structures and energies of the insertion intermediates

As the reaction proceeds, after PTSXH_n_, the intermediate PIMXH_n_ is formed with the breaking of X-H bond and the generation of Sn-X and Sn-H bonds. In PIMXH_n_, compared with PTSXH_n_, the X-H bond was extended to 2.900, 2.900, and 2.787 Å, respectively, indicating that it was gradually broken during the formation of intermediates. The Sn-X and Sn-H distances were shortened to 2.058, 2.004, 1.962 and 1.703, 1.706, and 1.697 Å, respectively, indicating that the Sn-X and Sn-H bonds were gradually formed. While the Sn-F and Sn-Li bonds were significantly prolonged, indicating that the LiF part was gradually separated. At this time, the natural charge of the Sn atom increases, and the natural charge of X atom and H atom decreased significantly, indicating that the nucleophilic reaction continued to occur. The relative energies of PIMXH_n_ are 41.58, 1.21, and −54.09 kJ/mol, respectively.

#### 3.1.4. The structures and energies of the products

As the reaction proceeds, when LiF is completely separated from Sn, the product PPXH_n_ is obtained. It can be found from Figure 2 that the structures of PPNH_3_, PPH_2_O and PPHF are very similar, and they are all in four-coordinate configuration. Their relative energies are 96.52, 68.74, and 28.96 kJ/mol, respectively, indicating that the insertion reaction of RP is endothermic.

As aforementioned, the insertion reaction of RP and XH_n_ is endothermic. The three products are similar. The order of the energy barrier is NH_3_ > H_2_O > HF, which indicates that HF is more prone to the insertion reaction, followed by H_2_O and NH_3_.

#### 3.1.5. Mechanism of the insertion reaction

In order to explain the mechanism of the insertion reaction of RP with XH_n_, we carried out the intrinsic reaction coordinate (IRC) analysis based on the optimized structure of transition states (PTSNH_3_, PTSH_2_O, PTSHF). Since the mechanism is similar, the reaction of RP with NH_3_ is chosen as an example to describe.

Figure 4 shows the changes of energy and Sn-H_a_, Sn-N and N-H_a_ bond distances along the reaction coordinates. It can be seen from the figure that in the region of the reaction coordinate (10–0), the energy rises sharply and reaches the maximum energy at 0 point, which is in the transition state (PTSNH_3_). In this region, the distance between Sn-H_a_ and Sn-N is continuously shortened, which means that NH_3_ is constantly approaching RP and has the tendency to form new bonds. The distance between N-H_a_ bonds began to lengthen, and H_a_ tended to leave NH_3_. After 0 point, the energy of the reaction system begins to decrease. The distance between Sn-H_a_ and Sn-N gradually decreases to a constant, which indicates that new Sn-H_a_ and Sn-N bonds have been formed, accompanied by the fracture of N-H_a_ bonds.

### 3.2. Insertion reaction of three-membered ring configuration (RS)

It can be seen from Figure 1 that the components of HOMO of RS are mainly concentrated on Sn atom and two H atoms, while the components of LUMO are mainly concentrated on Li atom, and a small part of them are concentrated on the p orbitals of Sn atom. Since the F atom gives electrons to the p orbital on the Sn atom, the p orbital on the Sn atom is not empty. When the insertion reaction occurs, the H end of the X-H bond first attacks the Sn atom σ orbit, and then close to RS, and the electron is partially transferred to the s orbital of H atom. Then, the X end of the X-H bond interacts with the p orbital on the Sn atom in RS to complete the reaction.

#### 3.2.1. The structures and energies of the precursor complexes

At the beginning of the reaction, X-H is close to RS, and the H end of X-H bond is connected with σ Orbital of Sn atom. The orbits combine to form the precursor SQ_x_. From the Figure 3, the distances between Sn-X and Sn-H are 2.601, 2.711, 2.996 Å and 3.149, 3.032, 2.677 Å respectively, and the relative energies of SQ_N_, SQ_O_ and SQ_F_ are–38.47, −23.56, and −16.25 kJ/mol respectively, which indicates that this is an exothermic process.

#### 3.2.2. The structures and energies of the transition states

As XH_n_ approaches RS further, the X terminal interacts with the p orbital on the Sn atom on the back of the F atom in RS. The interaction between XH_n_ and Sn atom weakens the X-H bond. The reaction reached the transition state STSXH_n_. As shown in the Figure 3, the Sn-H distance was 1.853, 1.843, 1.826 Å and the Sn-X distance was 2.259, 2.228, and 2.268 Å respectively. Compared with SQ_X_, the Sn-H and Sn-X distances were significantly shortened. It can be seen from the Tables (S4–S6) that the natural charge of sn atom increased from 1.522 to 2.305, 2.334, 2.317 respectively, and the natural charge of X and H atoms decreased significantly, indicating that this is a nucleophilic process. Frequency analysis calculations performed at the M06-2X/def2-TZVP level of theory show that STSX_n_ has a unique imaginary frequency (1582.0, 1527.1, 1426.3 i cm^−1^). IRC analysis shows that STSX_n_ is the real transition state in the insertion reaction of H_2_SnLiF with XH_n_, and correctly connects the precursor complex SQ_X_ and the intermediate SIMXH_n_. The relative energy of STSXH_n_ is 208.66, 165.09, 114.86 kJ/mol respectively, so the potential barrier of RS insertion reaction is 247.13, 188.65, 131.11 kJ/mol respectively.

#### 3.2.3. The structures and energies of the insertion intermediates

After the transition state STSXH_n_, as the reaction proceeds, the X-H bond gradually breaks, the Sn-H and Sn-X bonds gradually form, forming the intermediate SIMXH_n_. At this time, the natural charge of the Sn atom increases, the natural charge of the X and H atoms decreases, the electrons of XH_n_ attack the Sn atom as a nucleophilic reagent, the ternary ring in RS is destroyed, the distance between the Li atom and the F atom is shortened, and there is a tendency to leave. The relative energy of SIMXH_n_ is 6.69, −34.67 and −90.90 kJ/mol respectively.

#### 3.2.4. The structures and energies of the insertion products

As the reaction proceeds, LiF leaves and the product SPXH_n_ is obtained. It can be seen from the Figure 3 that the structures of SPNH_3_, SPH_2_O and SPHF are very similar, and are the same as the insertion reaction products of RP. Their relative energies are 59.71, 31.94, and −7.86 kJ/mol respectively, indicating that the insertion reaction of RP with NH_3_ and H_2_O is endothermic, and the reaction with HF is exothermic.

It can be seen from the above that the insertion reaction products of RS and XH_n_ are the same as those of RP reaction. The order of the insertion reaction barrier size is NH_3_ > H_2_O > HF, which is the same as that of RP reaction. And the reaction barrier of RS and RP are compared respectively. We find that the energy barrier overcome by RS is lower and the reaction is easier to occur.

#### 3.2.4. Mechanism of the Insertion reaction

In order to explain the mechanism of the insertion reaction of RS with NH_3_, H_2_O and HF, we carried out the intrinsic reaction coordinate (IRC) analysis based on the optimized structure of transition states (STSNH_3_, STSH_2_O, and STSHF). Taking the reaction with NH_3_ as an example, the reaction mechanism of RS and XH_n_ (X = N, O, F, n = 3, 2, 1) will be summarized below.

Figure 5 shows the change of reaction coordinates along the reaction path of Sn-H_d_, Sn-N and N-H_d_ distances with energy. In the reaction between RS and NH_3_, it can be seen from the figure that in the region of the reaction coordinate (20–0), the energy rises sharply and reaches the maximum energy at point 0. At this time, it is in the transition state (STSNH_3_). In this region, we can see that the distance between Sn-H_d_ and Sn-N is continuously shortened, which means that NH_3_ is constantly approaching RS, the distance between N-H_d_ bonds begins to lengthen, and H_d_ has a tendency to leave NH_3_. After 0, the energy of the system begins to decrease, and the system gradually becomes stable. The distance between Sn-H_d_ and Sn-N is shortened to a certain extent and becomes stable, which indicates that new Sn-H_d_ and Sn-N bonds have been formed. The distance between N-H_d_ bonds is extended to a certain extent. At this time, H_d_ atom has been combined with Sn atom and LiF has been separated.

### 3.3. Solventing effect

In order to study the effect of solvent on the insertion reaction, we use the SMD model to set the insertion reaction to occur in THF solvent. The calculated results show that for the reaction involving RP, the inserted reaction barrier in THF solvent is 263.11, 190.19, 121.97 kJ/mol, which is lower than the reaction barrier in vacuum. For the reaction involving RS, the inserted reaction barrier in THF solvent is 273.07, 203.28, 141.56 kJ/mol, which is increased compared with the reaction barrier under vacuum. This shows that for RS, the reaction in THF solvent is unfavorable. For RP and RS, the order of difficulty of insertion reaction in THF solvent is N > O > F, and the reaction barrier of RS is lower than that of RP, so it is easier to conduct insertion reaction.

## 4. Conclusion

In this paper, the insertion reactions of the p-complex configuration (RP) and the ternary ring configuration (RS) of stannylenoid H_2_SnLiF with NH_3_, H_2_O, and HF have been studied theoretically. According to the calculation results, we know that the reaction products of RP and RS are the same, and through the calculation of the reaction energy barrier, the insertion reaction potential barrier of RS is lower than that of RP, which means that RS is easier to react. Comparing the reaction barrier of RP and RS with NH_3_, H_2_O, and HF, we found that the difficulty of insertion reaction is NH_3_ > H_2_O > HF, regardless of the configuration of p-complex or three-membered ring. According to the calculation results, in THF solvent, the insertion reaction of RP with NH_3_, H_2_O, and HF is favorable, while the insertion reaction of RS with NH_3_, H_2_O, and HF is unfavorable. In addition, for RP and RS, the difficulty order of insertion reaction in THF solvent is N > O > F, and the reaction barrier of RS is lower than that of RP, so it is easier to conduct insertion reaction. We hope that the calculation results given in this paper are satisfactory and can make some useful predictions for the experiment.

## Supplementary Data

**Table S1:** Natural bond orbital analysis (NBO) of the stagnation points on the potential energy surface of RP and NH_3_ insertion reaction at M06-2X/def2-TZVP level.

Structure	Sn	F	Li	H	H	N	H _a_	H _N1_	H _N2_
RP	1.438	−0.884	0.764	−0.659	−0.659				
NH _3_						−1.053	0.351	0.351	0.351
LiF		0.923	−0.923						
PTSNH _3_	2.242	−0.900	0.861	−0.577	−0.687	−1.446	−0.215	0.363	0.358
PIMNH _3_	2.696	−0.907	0.875	−0.571	−0.682	−1.554	−0.571	0.361	0.361
PPNH _3_	2.554			−0.581	−0.581	−1.538	−0.592	0.369	0.369

(Tips: H_N1_ and H_N2_ are atoms connected with N atom).

**Table S2:** Natural bond orbital analysis (NBO) of the stagnation points on the potential energy surface of RS and H_2_O insertion reaction at M06-2X/def2-TZVP level.

Structure	Sn	F	Li	H	H	O	H _b_	H _o_
RP	1.438	−0.884	0.764	−0.659	−0.659			
H _2_O						−0.919	0.459	0.459
LiF		0.923	−0.923					
PTSH _2_O	2.288	−0.899	0.869	−0.589	−0.683	−1.257	−0.198	0.469
PIMH _2_O	2.724	−0.907	0.883	−0.582	−0.688	−1.319	−0.569	0.460
PPH _2_O	2.589			−0.589	−0.589	−1.302	−0.577	0.470

(Tips: H_o_ are atoms connected with O atom).

**Table S3:** Natural bond orbital analysis (NBO) of the stagnation points on the potential energy surface of RP and HF insertion reaction at M06-2X/def2-TZVP level.

Structure	Sn	F	Li	H	H	^*^F	H _f_
RP	1.438	−0.884	0.764	−0.659	−0.659		
HF						−0.550	0.550
LiF		0.923	−0.923				
PTSHF	2.288	−0.898	0.877	−0.685	−0.579	−0.812	−0.190
PIMHF	2.732	−0.907	0.890	−0.575	−0.575	−0.881	−0.684
PPHF	2.603			−0.581	−0.581	−0.859	−0.582

(Tips: H_f_ are atoms connected with ^*^F atom).

**Table S4:** Natural bond orbital analysis (NBO) of the stagnation points on the potential energy surface of RS and NH_3_ insertion reaction at M06-2X/def2-TZVP level.

Structure	Sn	F	Li	H	H	N	H _d_	H _N1_	H _N2_
RS	1.522	−0.894	0.588	−0.608	−0.608				
NH _3_						−1.053	0.351	0.351	0.351
LiF		0.923	−0.923						
STSNH _3_	2.305	−0.897	0.676	−0.574	−0.574	−1.454	−0.220	0.368	0.371
SIMNH _3_	2.694	−0.907	0.880	−0.578	−0.574	−1.561	−0.680	0.362	0.364
SPNH _3_	2.554			−0.581	−0.581	−1.538	−0.592	0.369	0.369

(Tips: H_N1_ and H_N2_ are atoms connected with N atom).

**Table S5:** Natural bond orbital analysis (NBO) of the stagnation points on the potential energy surface of RS and H_2_O insertion reaction at M06-2X/def2-TZVP level.

Structure	Sn	F	Li	H	H	O	H _e_	H _o_
RS	1.522	−0.894	0.588	−0.608	−0.608			
H _2_O						−0.919	0.459	0.459
LiF		0.923	−0.923					
STSH _2_O	2.334	−0.896	0.692	−0.583	−0.571	−1.245	−0.201	0.472
SIMH _2_O	2.723	−0.907	0.887	−0.582	−0.582	−1.321	−0.678	0.460
SPH _2_O	2.589			−0.590	−0.590	−1.302	−0.577	0.470

(Tips: H_o_ are atoms connected with O atom).

**Table S6:** Natural bond orbital analysis (NBO) of the stagnation points on the potential energy surface of RS and HF insertion reaction at M06-2X/def2-TZVP level.

Structure	Sn	F	Li	H	H	^*^F	H _f_
RS	1.522	−0.894	0.588	−0.608	−0.608		
HF						−0.550	0.550
LiF		0.923	−0.923				
STSHF	2.317	−0.897	0.722	−0.572	−0.572	−0.803	−0.194
SIMHF	2.732	−0.907	0.890	−0.575	−0.575	−0.881	−0.684
SPHF	2.603			−0.581	−0.581	−0.859	−0.582

(Tips: H_f_ are atoms connected with ^*^F atom).

**Table S7:** Energy of each stationary point on the potential energy surface of RP insertion reaction (kJ/mol).

Structure	Energy (vacuum)/a.u.	Energy (THF)/a.u.
RP	−322.1117	−322.1550
NH _3_	−56.4575	−56.4621
H _2_O	−76.3200	−76.3262
HF	−100.3336	−100.3385
QPNH _3_	−378.5805	−378.6236
QPH _2_O	−398.4382	−398.4839
OPHF	−422.4513	−422.5025
TSPNH _3_	−378.4751	−378.5233
TSPH _2_O	−398.3550	−398.4115
TSPHF	−422.3892	−422.4560
IMPNH _3_	−378.5535	−378.6029
IMPH _2_O	−398.4313	−398.4857
IMPHF	−422.4659	−422.5249
PPNH _3_	−271.2763	−271.2805
PPH _2_O	−291.1493	−291.1551
PPHF	−315.1780	−315.1843
LiF	−107.2563	−107.3095

**Table S8:** Energy of each stationary point on the potential energy surface of RS insertion reaction (kJ/mol).

Structure	Energy (vacuum)/a.u.	Energy (THF)/a.u.
RS	−322.0977	−322.1262
QSNH _3_	−378.5699	−378.5966
QSH _2_O	−398.4267	−398.4522
OSHF	−422.4375	−422.4629
TSSNH _3_	−378.4758	−378.4925
TSSH _2_O	−398.3549	−398.3755
TSSHF	−422.3875	−422.4133
IMSNH _3_	−378.5527	−378.5752
IMSH _2_O	−398.4309	−398.4558
IMSHF	−422.4659	−422.4936
PSNH _3_	−271.2763	−271.2796
PSH _2_O	−291.1493	−291.1543
PSHF	−315.1780	−315.1850
